# Supplementary material for: Extended Sentinel Monitoring of Helicoverpa zea Resistance to Cry and Vip3Aa Toxins in Bt Sweet Corn: Assessing Changes in Phenotypic and Allele Frequencies of Resistance
Source: Insects. 2023 Jun 25;14(7):577. doi: 10.3390/insects14070577 (PMC10380249; doi:10.3390/insects14070577)
Supplement: Supplementary file 1 [file insects-14-00577-s001.zip › Table S1.pdf]

Table S1. Percentages of non-Bt ears damaged by *H. zea*, *O. nubilalis, S. frugiperda* and *S. albicosta* at each sentinel trial during each year of the sweet corn sentinel monitoring network. Data were averaged over the non-Bt hybrids ('Providence' and 'Obsession I') and listed according to the highest to lowest means of *H. zea* damage.

Year	Trial location	*H. zea*	*O. nubilalis*	*S. frugiperda*	*S. albicosta*
2020	Salisbury, MD	100.0	0.0	0.0	0.0
	Queenstown, MD	100.0	2.0	0.0	0.0
	Brenton, AL	100.0	0.0	0.0	0.0
	Suffolk, VA	100.0	0.0	1.0	0.0
	Plains, GA	100.0	0.0	3.0	0.0
	Stoneville1, MS	100.0	0.0	0.0	0.0
	Lubbock, TX	98.8	1.3	3.3	0.0
	Beltsville3, MD	98.0	0.0	2.0	0.0
	Ames, IA	98.0	0.0	5.0	0.0
	RAREC, NJ	98.0	0.0	0.0	0.0
	Griffin, GA	98.0	0.0	0.0	0.0
	Stoneville2, MS	98.0	0.0	0.0	0.0
	Jackson City, NC	98.0	0.0	0.0	0.0
	Plymouth, NC	97.0	0.0	1.0	0.0
	Abingdon, VA	97.0	0.0	0.0	0.0
	Beltsville4, MD	96.1	0.0	0.0	0.0
	Riverhead, NY	95.5	5.5	0.0	0.0
	Painter, VA	94.3	2.0	45.0	0.0
	Champaign, IL	94.0	3.0	4.0	0.0
	Catawba, VA	92.0	0.0	0.0	0.0
	Keedysville, MD	90.0	2.0	0.0	0.0
	Tallassee, Al	89.5	0.0	0.0	0.0
	Jay, FL	89.0	0.0	0.0	0.0
	Whitethome, VA	88.1	0.0	0.0	0.0
	Clay Center, NE	87.7	0.0	0.0	0.0
	Pittstown, NJ	86.5	3.5	0.0	0.0
	Florence, SC	85.0	0.0	53.0	0.0
	Stoneville3, MS	85.0	0.0	0.0	0.0
	Georgetown, DE	84.4	0.6	0.0	0.0

	Rosemont, MN	79.0	0.0	0.0	0.0
	Queenstown, MD	70.5	0.0	0.0	0.0
	Rock Springs, PA	64.5	18.0	1.0	0.0
	Berwick, NS	39.0	2.0	0.0	8.0
	Lafayette, IN	36.0	0.0	11.0	0.0
	Freetown, PEI	18.4	0.0	1.0	0.0
	Arlington, WI	16.5	0.0	0.0	0.0
	Ridgetown, ON	15.5	0.0	1.0	0.0
	S. Charleston, OH	7.5	0.0	6.5	0.0
	Frankenmuth, MI	0.0	0.0	0.0	2.5
	Ridgetown, ON	0.0	0.0	0.0	1.0
	St. Mathieude, QC	0.0	0.0	0.0	5.5
2021	Beltsville1, MD	100.0	0.0	0.0	0.0
	Beltsville2, MD	100.0	0.0	0.0	0.0
	Queenstown1, MD	100.0	0.0	0.0	0.0
	Queenstown2, MD	100.0	0.0	0.0	0.0
	Stoneville2, MS	100.0	0.0	0.0	0.0
	Stoneville3, MS	100.0	0.0	3.0	0.0
	Lubbock, TX	100.0	0.0	16.0	0.0
	Plymouth2, NC	100.0	0.0	0.0	0.0
	Lafayette, IN	99.5	4.5	0.0	0.0
	S. Charleston, OH	99.0	0.0	0.0	0.0
	Champaign, IL	99.0	0.0	0.0	0.0
	Suffolk, VA	99.0	0.0	0.0	0.0
	Florence, SC	98.0	0.0	3.0	0.0
	Painter, VA	97.5	8.5	5.5	0.0
	RAREC, NJ	96.0	0.0	2.0	0.0
	Ames, IA	96.0	0.0	0.0	0.0
	Georgetown, DE	94.5	0.0	0.0	0.0
	Rosemount, MN	93.0	0.0	0.0	0.0
	Griffin, GA	91.5	0.0	0.0	0.0
	Red Rock, AZ	90.2	0.0	3.9	0.0

	Jay, FL	87.0	0.0	4.7	0.0
	Wooster, OH	86.0	2.0	0.0	0.0
	Brewton, AL	85.7	0.0	0.0	0.0
	Pittstown, NJ	85.0	5.0	0.0	0.0
	Salisbury, MD	83.9	0.0	0.0	0.0
	Shorter, AL	82.3	0.0	0.0	0.0
	Corpus Christi2, TX	81.8	0.0	20.4	0.0
	Stoneville1, MS	79.0	0.0	0.0	0.0
	Clay Center, NE	78.7	0.0	0.0	0.0
	Abingdin, VA	78.5	0.0	0.0	0.0
	Winnsboro, LA	70.5	0.0	0.0	0.0
	Keedysville, MD	67.5	4.3	0.0	0.0
	Corpus Christi1, TX	66.4	0.0	8.2	0.0
	Rock Springs, PA	64.0	12.0	0.0	0.0
	Lewiston, NC	61.4	0.0	0.0	0.0
	Jackson Spr, NC	54.9	0.0	0.0	0.0
	Winnsboro, LA	42.0	0.0	0.0	0.0
	Whitehome, VA	40.3	0.0	0.0	0.0
	Geneva, NY	32.0	0.0	0.0	0.0
	Cambridge1, NS	32.0	0.0	0.0	0.0
	Durham, NH	30.0	0.0	0.0	0.0
	Cambridge2, NS	26.0	0.0	0.0	0.0
	Arlington, WI	22.0	0.0	0.0	0.0
	Emerald, PEI	14.0	0.0	3.0	0.0
	Plymouth1, NC	11.9	0.0	0.0	0.0
	Plains, GA	6.5	0.0	0.0	0.0
	Ridgetown, ON	4.0	0.0	1.5	59.5
	Frankenmuth, MI	3.0	0.8	0.0	0.0
	N. Platte, NE	1.9	0.0	0.0	27.1
	Ridgetown, ON	1.0	0.0	1.0	14.0
	St. Mathieu, QC	0.0	0.0	0.0	5.5

	Winchester, ON	0.0	27.5	5.0	0.0
2022	Beltsville2, MD	100.0	0.0	0.0	0.0
	Queenstown, MD	100.0	0.0	0.0	0.0
	Salisbury, MD	100.0	0.0	0.0	0.0
	Champaign, IL	100.0	0.0	1.0	0.0
	Florence, SC	100.0	0.0	0.0	0.0
	Stoneville, MS	100.0	0.0	0.0	0.0
	Stoneville, MS	100.0	0.0	0.0	0.0
	Jackson Spr1, NC	100.0	0.0	0.0	0.0
	Suffolk, VA	100.0	0.0	0.0	0.0
	RAREC, NJ	100.0	0.0	0.0	0.0
	Riverhead, NY	99.5	2.9	0.0	0.0
	Abington, VA	99.5	0.0	0.0	0.0
	Beltsville1, MD	99.0	0.0	0.0	0.0
	Painter, VA	99.0	1.0	9.0	0.0
	Whitethome, VA	99.0	0.0	2.5	0.0
	Lubbock, TX	99.0	0.0	20.0	0.0
	Pittstown, NJ	97.0	0.0	0.0	0.0
	Georgetown, DE	96.0	0.0	0.0	0.0
	Winnsboro, LA	95.5	0.0	0.0	0.0
	Griffin, GA	95.0	0.0	0.0	0.0
	Brewton, AL	95.0	0.0	2.7	0.0
	Ames, IA	94.1	0.0	0.0	0.0
	Geneva, NY	94.0	10.0	2.0	0.0
	Dean Lee, LA	94.0	0.0	1.0	0.0
	Charleston, SC	94.0	0.0	0.0	0.0
	Keedysville, MD	92.2	2.0	0.0	0.0
	Stoneville, MS	91.0	0.0	0.0	0.0
	Newark, DE	90.6	0.0	0.0	0.0
	Plains, GA	90.5	0.0	0.0	0.0

	TPAC, IN	90.5	0.0	0.0	0.0
	Plymouth, NC	88.0	0.0	0.0	0.0
	Corpus Christi, TX	87.9	0.0	5.5	0.0
	Jackson Spr2, NC	87.2	0.0	0.0	0.0
	Maricopa, AZ	87.0	0.0	5.7	0.0
	Red Rock, AZ	86.7	0.0	5.0	0.0
	Shorter, Al	81.3	0.0	0.0	0.0
	Westaco, TX	79.3	0.0	0.0	0.0
	BREC, MD	78.9	0.0	0.0	0.0
	Brewton, AL	67.0	0.0	6.6	0.0
	Harvard, NE	64.9	0.0	0.0	14.5
	BREC, MD	64.0	0.0	0.0	0.0
	Kentville, NS	46.0	4.0	0.0	21.0
	S. Charleston, OH	45.0	3.0	0.0	0.0
	Lancaster, PA	34.0	1.0	0.0	0.0
	Wooster, OH	33.0	2.0	0.0	0.0
	Plymouth, NC	23.0	0.0	0.0	0.0
	Sussex, NB	17.0	0.0	1.0	26.0
	Alburgh, VT	16.0	0.5	0.0	0.0
	Ridgetown, ON	13.0	1.0	4.0	1.0
	Brookings, SD	5.0	0.0	0.0	0.0
	Frankenmuth, MI	5.0	3.5	2.5	0.0
	St. Mathieu, QC	0.0	4.5	0.0	24.5
	Frankenmuth, MI	0.0	0.0	0.0	2.0
