# Supplementary material for: Extended Sentinel Monitoring of Helicoverpa zea Resistance to Cry and Vip3Aa Toxins in Bt Sweet Corn: Assessing Changes in Phenotypic and Allele Frequencies of Resistance
Source: Insects. 2023 Jun 25;14(7):577. doi: 10.3390/insects14070577 (PMC10380249; doi:10.3390/insects14070577)
Supplement: Supplementary file 1 [file insects-14-00577-s001.zip › Table S2.pdf]

Table S2. Percentage of non-Bt ears damaged by *H. zea* and the estimated phenotypic frequency of resistance for each Bt toxin per sentinel trial during each year of the 2020-2022 sweet corn sentinel monitoring network. Data were averaged over the non-Bt hybrids ('Providence' and 'Obsession I') and listed according to the highest to lowest means of *H. zea* damage by year. PFR is the ratio of mean density of surviving *H. zea* larvae per Bt ear relative to the mean surviving larval density per non-Bt ear.

Year	Trial location	% non-Bt ears damaged by *H. zea*	Phenotypic Frequency of Resistance (PFR)		
			Cry1Ab	Cry1A.105/ Cry2Ab2	VipAa
2020	Salisbury, MD	100.0	0.61	0.73	0.0
	Queenstown, MD	100.0	0.79	0.65	0.0
	Brenton, AL	100.0	0.65	0.52	0.0
	Suffolk, VA	100.0	NA	0.91	0.0
	Plains, GA	100.0	NA	NA	0.0
	Stoneville1, MS	100.0	1.07	1.06	0.0
	Lubbock, TX	98.8	1.10	NA	0.0
	Beltsville3, MD	98.0	1.62	0.68	0.0
	Ames, IA	98.0	1.98	NA	0.0
	RAREC, NJ	98.0	0.79	0.91	0.0
	Griffin, GA	98.0	1.00	0.98	0.0
	Stoneville2, MS	98.0	1.19	NA	0. 0559
	Jackson City, NC	98.0	0.94	1.09	0.0
	Plymouth, NC	97.0	NA	0.79	0.0
	Abingdon, VA	97.0	0.91	1.06	0.0
	Beltsville4, MD	96.1	0.53	1.16	0.0
	Riverhead, NY	95.5	1.18	0.87	0.0051
	Painter, VA	94.3	0.75	1.26	0.0
	Champaign, IL	94.0	1.11	1.47	0.0
	Catawba, VA	92.0	1.43	NA	0.0
	Keedysville, MD	90.0	0.61	0.70	0.0
	Tallassee, Al	89.5	0.92	0.51	0.0
	Jay, FL	89.0	0.98	1.03	0.0
	Whitethome, VA	88.1	NA	NA	0.0
	Clay Center, NE	87.7	0.92	0.86	0.0
	Pittstown, NJ	86.5	1.05	NA	0.0

	Florence, SC	85.0	0.88	0.55	0.0
	Stoneville3, MS	85.0	1.11	1.03	0.0
	Georgetown, DE	84.4	0.86	NA	0.0
	Rosemont, MN	79.0	0.83	NA	0.0
	Queenstown, MD	70.5	0.74	0.79	0.0
	Rock Springs, PA	64.5	0.41	0.50	0.0
	Berwick, NS	39.0	NA	NA	NA
	Lafayette, IN	36.0	0.89	NA	NA
	Freetown, PEI	18.4	NA	NA	NA
	Arlington, WI	16.5	NA	NA	NA
	Ridgetown, ON	15.5	NA	NA	NA
	S. Charleston, OH	7.5	NA	NA	NA
	Frankenmuth, MI	0.0	NA	NA	NA
	Ridgetown, ON	0.0	NA	NA	NA
	St. Mathieude, QC	0.0	NA	NA	NA
2021	Beltsville1, MD	100.0	1.34	0.97	0.0031
	Beltsville2, MD	100.0	1.06	0.74	0.0056
	Queenstown1, MD	100.0	1.67	1.11	0.0
	Queenstown2, MD	100.0	1.03	1.07	0.0
	Stoneville2, MS	100.0	1.02	0.91	0.0
	Stoneville3, MS	100.0	1.01	0.71	0.0
	Lubbock, TX	100.0	NA	NA	0.0
	Plymouth2, NC	100.0	NA	NA	0.0
	Lafayette, IN	99.5	0.90	0.62	0.0127
	S. Charleston, OH	99.0	0.91	NA	0.0046
	Champaign, IL	99.0	1.05	1.11	0.0
	Suffolk, VA	99.0	NA	NA	0.0
	Florence, SC	98.0	0.75	0.65	0.0
	Painter, VA	97.5	1.04	0.93	0.0234
	RAREC, NJ	96.0	1.00	1.03	0.0
	Ames, IA	96.0	1.00	0.86	0.0023
	Georgetown, DE	94.5	1.02	1.39	0.0

	Rosemount, MN	93.0	0.90	0.94	0.0
	Griffin, GA	91.5	1.19	NA	0.0043
	Red Rock, AZ	90.2	NA	NA	0.0
	Jay, FL	87.0	1.07	1.00	0.0595
	Wooster, OH	86.0	NA	NA	0.0
	Brewton, AL	85.7	1.09	0.63	0.0063
	Pittstown, NJ	85.0	0.93	NA	0.0
	Salisbury, MD	83.9	0.83	0.52	0.0
	Shorter, AL	82.3	1.11	0.59	0.0
	Corpus Christi2, TX	81.8	1.19	2.22	0.0677
	Stoneville1, MS	79.0	0.59	0.97	0.0172
	Clay Center, NE	78.7	1.14	0.69	0.0098
	Abingdin, VA	78.5	NA	NA	0.0
	Winnsboro, LA	70.5	NA	NA	0.0
	Keedysville, MD	67.5	0.54	NA	0.0
	Corpus Christi1, TX	66.4	0.28	1.21	0.0681
	Rock Springs, PA	64.0	0.87	0.57	0.0306
	Lewiston, NC	61.4	0.89	NA	0.0
	Jackson Spr, NC	54.9	0.87	0.57	0.0306
	Winnsboro, LA	42.0	NA	NA	0.0
	Whitehome, VA	40.3	NA	NA	0.0
	Geneva, NY	32.0	NA	NA	NA
	Cambridge1, NS	32.0	NA	NA	NA
	Durham, NH	30.0	NA	NA	NA
	Cambridge2, NS	26.0	NA	NA	NA
	Arlington, WI	22.0	NA	NA	NA
	Emerald, PEI	14.0	NA	NA	NA
	Plymouth1, NC	11.9	NA	NA	NA
	Plains, GA	6.5	NA	NA	NA
	Ridgetown, ON	4.0	NA	NA	NA
	Frankenmuth, MI	3.0	NA	0.0	NA

	N. Platte, NE	1.9	NA	NA	NA
	Ridgetown, ON	1.0	NA	NA	NA
	St. Mathieu, QC	0.0	NA	NA	NA
	Winchester, ON	0.0	NA	NA	NA
2022	Beltsville2, MD	100.0	1.30	0.67	0.0
	Queenstown, MD	100.0	0.96	0.77	0.0
	Salisbury, MD	100.0	1.11	0.92	0.0
	Champaign, IL	100.0	1.21	1.42	0.0
	Florence, SC	100.0	1.09	0.84	0.0
	Stoneville, MS	100.0	1.19	0.79	0.0
	Stoneville, MS	100.0	0.81	1.04	0.0
	Jackson Spr1, NC	100.0	1.38	0.93	0.0423
	Suffolk, VA	100.0	1.01	0.96	0.0053
	RAREC, NJ	100.0	NA	NA	0.0
	Riverhead, NY	99.5	1.01	0.96	0.0053
	Abington, VA	99.5	1.20	0.82	0.0049
	Beltsville1, MD	99.0	1.42	0.69	0.0
	Painter, VA	99.0	0.87	0.83	0.0
	Whitethome, VA	99.0	0.95	0.77	0.0029
	Lubbock, TX	99.0	1.50	1.40	0.0
	Pittstown, NJ	97.0	0.90	1.00	0.0
	Georgetown, DE	96.0	0.77	0.59	0.0
	Winnsboro, LA	95.5	1.06	0.61	0.0
	Griffin, GA	95.0	0.91	0.79	0.0
	Brewton, AL	95.0	0.84	0.37	0.0
	Ames, IA	94.1	1.19	0.84	0.0
	Geneva, NY	94.0	0.76	0.78	0.0
	Dean Lee, LA	94.0	0.92	0.41	0.0
	Charleston, SC	94.0	NA	NA	0.0
	Keedysville, MD	92.2	1.02	0.73	0.0086

	Stoneville, MS	91.0	0.97	0.93	0.0
	Newark, DE	90.6	0.91	0.85	0.0074
	Plains, GA	90.5	0.91	0.94	0.0
	TPAC, IN	90.5	0.71	0.60	0.0054
	Plymouth, NC	88.0	1.07	0.67	0.0
	Corpus Christi, TX	87.9	1.03	0.73	0.0
	Jackson Spr2, NC	87.2	1.08	1.11	0.0
	Maricopa, AZ	87.0	NA	NA	0.0
	Red Rock, AZ	86.7	NA	NA	0.0
	Shorter, Al	81.3	0.46	NA	0.0149
	Westaco, TX	79.3	NA	NA	0.0
	BREC, MD	78.9	NA	NA	0.0
	Brewton, AL	67.0	1.09	NA	0.0
	Harvard, NE	64.9	1.10	0.74	0.0
	BREC, MD	64.0	NA	NA	0.0
	Kentville, NS	46.0	NA	NA	NA
	S. Charleston, OH	45.0	NA	NA	NA
	Lancaster, PA	34.0	NA	NA	NA
	Wooster, OH	33.0	NA	NA	NA
	Plymouth, NC	23.0	NA	NA	NA
	Sussex, NB	17.0	NA	NA	NA
	Alburgh, VT	16.0	NA	NA	NA
	Ridgetown, ON	13.0	NA	NA	NA
	Brookings, SD	5.0	NA	NA	NA
	Frankenmuth, MI	5.0	NA	NA	NA
	St. Mathieu, QC	0.0	NA	NA	NA
	Frankenmuth, MI	0.0	NA	NA	NA

NA = data not available due to low *H. zea* infestation and/or sampling timed too early to estimate the maximum number of surviving larvae.
